# *Shigella dysenteriae* Modulates BMP Pathway to Induce Mucin Gene Expression *In Vivo* and *In Vitro*

**DOI:** 10.1371/journal.pone.0111408

**Published:** 2014-11-03

**Authors:** Ashidha Gopal, Soumya Chidambaram Iyer, Udhayakumar Gopal, Niranjali Devaraj, Devaraj Halagowder

**Affiliations:** 1 Unit of Biochemistry, Department of Zoology, School of Life Sciences, University of Madras, Guindy Campus, Chennai, Tamilnadu, India; 2 Department of Pathology, Duke University Medical Center, Durham, North Carolina, United States of America; 3 Department of Biochemistry, University of Madras, Guindy Campus, Chennai, Tamilnadu, India; University of Nebraska Medical Center, United States of America

## Abstract

Mucosal epithelial cells in the intestine act as the first line of host defense against pathogens by increasing mucin production for clearance. Despite this fact, the underlying molecular mechanisms by which *Shigella dysenteriae* transduce mucin gene expression remain poorly defined. The goal of this study was to determine the role of Bone morphogenetic protein (BMP) pathway in mucin gene expression during *S. dysenteriae* infection. In this study we demonstrate that *S. dysenteriae* activates BMP signaling to induce MUC2 and MUC5AC gene expression in rat ileal loop model and *in vitro*. We also observed that BMP pathway regulates CDX2 expression which plays a critical role in induction of MUC2 gene during *S. dysenteriae* infection. In SMAD4 silenced cells *S. dysenteriae* infection did not abrogate MUC2 and MUC5AC gene expression whereas in CDX2 silenced cells it induces differential expression of MUC5AC gene. These results suggest that SMAD4-CDX2 induces MUC2 gene expression whereas SMAD4 directly influences differential expression of MUC5AC gene. Altogether, our results show that during *S. dysenteriae* infection the BMP pathway modulates inflammatory transcription factors CDX2 and SMAD4 to induce MUC2 and MUC5AC gene expression which plays a key role in the regulation of host mucosal defense thereby paving a cue for therapeutic application.

## Introduction

The Gram-negative invasive bacterial pathogen *Shigella* causes dysentery which is characterized by the induction of acute inflammation, thereby responsible for the massive tissue destruction of the intestinal mucosa [1]. The mucus layer that forms the primary barrier against pathogenic infections comprise mainly of mucins produced by goblet cells that enables the host to inhibit the access of pathogens to the underlying mucosa [2], [3]. In the adult intestine, MUC2 is the major gel-forming mucin stored within the goblet cells [3]. The importance of the mucus barrier is underlined by recent reports that mice deficient in MUC2 develop severe, life-threatening disease when infected with the attaching and effacing *Escherichia coli*-like pathogen *Citrobacter rodentium* and show delayed clearance of the nematode parasite *Trichuris muris* [4], [5]. It shows that in the intestine bacterial infection potentiate more severe pathology in the absence of secreted mucin MUC2. Another gel forming mucin MUC5AC, normally expressed in non-intestinal mucosa, has been reported to be expressed in the intestine, along with MUC2, during inflammation in diseases such as ulcerative colitis and adenocarcinoma and report state that MUC5AC acts as a direct and critical mediator of resistance during intestinal nematode infection [6], [7]. In our laboratory, we have shown previously that *S. dysenteriae* infection altered MUC2 and MUC5AC expression in rabbit ileal loop model and also demonstrated that Interleukin-1β and AKT signaling crosstalk to induce differential MUC5AC expression [8], [9]. However, the precise molecular mechanism underlying *S. dysenteriae* induced MUC2 and MUC5AC transcription remains obscure.

The Bone morphogenetic protiens (BMP's) are soluble proteins that are part of the transforming growth factor-β (TGF-β) superfamily exert pleiotropic biological effects, ranging from regulation of early development process to organogenesis and also promotes inflammation in response to bacterial infection in mice [10]–[12]. BMP ligands bind to a complex of the BMP receptor type II and a BMP receptor type I (Ia or Ib). This leads to the phosphorylation of the type I receptor that subsequently phosphorylates the BMP-specific SMADs (SMAD1, SMAD5 and SMAD8), allowing these receptor-associated SMADs to form a complex with SMAD4 and move into the nucleus where the SMAD complex binds a DNA binding protein and acts as a transcriptional enhancer [13]. Recent studies have suggested that bacterial infection promotes BMP pathway activation to induce CDX2 expression [12]. Caudal related homeobox gene CDX2 is an intestinal specific transcription factor which is essential for the intestinal development by regulating expression of intestinal genes like alkaline phosphatase, lactase and MUC2 [14]. However, so far it remains unclear whether *S. dysenteriae* regulates the transcription of MUC2 and MUC5AC. With this conception, it is of most important to know the mechanism that regulates CDX2 to induce mucin gene during *S. dysenteriae* infection.

Our aim is to understand the transcriptional regulation of mucin genes and their role during pathogenic infection that will help in proposing new therapeutic targets. Here we hypothesize that whether *S. dysenteriae* infection activates BMP pathway to regulate transcriptional expression of MUC2 and MUC5AC gene. In this study we show that SMAD4 regulates MUC2 expression through CDX2 transcription factor whereas it directly influences differential expression of MUC5AC gene. In conclusion we show for the first time that MUC2 and MUC5AC is upregulated at the transcriptional level by BMP pathway during *S. dysenteriae* infection which will be beneficial for the host.

## Materials and Methods

### Bacterial strains, growth condition and rat ileal loop infection with *S. dysenteriae*

Clinical isolates of *S. dysenteriae* were obtained from Department of Medical Microbiology, Christian Medical College (CMC), Vellore, India. The strains were routinely grown in Luria- Bertani (LB) broth (Himedia, Mumbai, India) at 37°C overnight. Wistar strain male albino rats weighing 120–150 g obtained from TANUVAS, Madhavaram, Chennai. The protocol was approved by the Institutional Animals Ethics Committee (IAEC) of University of Madras, INDIA, (approval no IAEC No. 011/02/2011). This study was carried out in accordance with the guidelines of the Committee for the Purpose of Control and Supervision on Experiments on Animals (CPCSEA). The rat ileal loop ligation assay was carried out by the method as previously described [15]. Male Wistar albino rats were fasted for 24 hr prior to experimentation and the animals were anaesthetized with Ketamine/Xylazine (90/10 mg/kg body weight). A Small midline incision (1–5 cm) was made down the abdomen exposing intestine. Loops were formed in the intestine with the silk thread at the ileocaecal junction. Inocula of 10^9^ CFU in 0.5 ml of Phosphate buffered saline (PBS), pH 7.4, were injected into ligated ileal loops. A total of nine male Wistar rats were used. Three male Wistar rats in each group were infected with *S. dysenteriae* at ileal section, allowed to live and sacrificed at 4 hr and 8 hr. The three non-infected loops with PBS served as control. The infected loops were used for immunohistochemistry, Western blot and PCR analysis.

### Cell culture and maintenance

HT29 human colon tumor cell line was obtained from National Centre for Cell Science, Pune, India. HT29 cells were grown in Dulbecco's Modified Eagle Medium (DMEM, GIBCO BRL, Germany), supplemented with 10% Fetal bovine serum (FBS) (Sigma, USA), 100 units/ml Penicillin, 100 mg/ml Streptomycin and 10–20 mg/ml fungisone (Himedia, India), pH 7.4 at 37°C under 5% CO_2_ and 95% air.

### Infection of HT29 cells with *S. dysenteriae*

HT29 cells were seeded into 6 or 12 well tissue culture plates at a density of 2×10^5^ cells/ml in volumes of 2 ml per well. At this seeding density, monolayers were sub-confluent (80–90%) at the time of the experiment. Bacteria were grown in LB medium overnight at 37°C and pelleted by centrifugation at 12,000 g for 5 min at 4°C. The pellets were washed with PBS (pH 7.4) twice and suspended in antibiotic-free DMEM. Bacteria at 100 cells per epithelial cell (100∶1 ratio) were used to infect for 2 hr to allow bacterial entry to occur. Monolayers were washed twice to remove extracellular bacteria and the cultures were incubated in the presence of 50 mg/ml of gentamicin to kill the remaining extracellular bacteria.

### BMP pathway activation

To activate BMP pathway, HT29 cells were treated for 24 hr with BMP2 (Sigma Aldrich, India) added to the culture medium, at a concentration of 50 or 100 ng/mL. Vehicle solution [4 mM HCl, 0.1% bovine serum albumin (BSA)] was used as the negative control.

### siRNA transfection

SMAD4 and CDX2 were silenced by transfection with SMAD4 siRNA and CDX2 siRNA (Santa Cruz Biotechnology, Santa Cruz, CA, USA). In a six-well tissue culture plate, 2×10^5^ cells per well were seeded in 2 ml antibiotic-free normal growth medium supplemented with FBS. Cells were incubated at 37°C in a CO_2_ incubator until the cells were 60–80% confluent. For each transfection, 4 µl of siRNA duplex which gives final concentration of 80 nM siRNA (Stock 20 µM siRNA) in 100 µl siRNA transfection medium (solution A) and 6 µl of siRNA transfection reagent in 100 µl siRNA transfection medium (solution B) were mixed and incubated for 45 min at room temperature. For each transfection, 0.8 ml siRNA transfection medium was added to each tube, mixed gently, overlaid onto washed cells and incubated for 5–7 hr at 37°C in a CO_2_ incubator. After incubation, 1 ml of normal growth medium containing twice the normal serum and antibiotic concentration (2× normal growth medium) was added without removing the transfection mixture. Cells were incubated for an additional 18–24 hr. Medium was replaced with 1 ml of fresh 1 x normal growth medium and used for further studies.

### Protein extraction and Western blot

Protein extraction from rat ileal tissue sample was performed by homogenizing in lysis buffer. The homogenate was centrifuged at 7,500 rpm at 4°C for 15 min and the supernatant was collected. The protein concentrations were determined by Lowry et al [16]. Cells were lysed and the protein content was measured using standard methods. Protein extracts (40–50 µg) were analysed by standard SDS-PAGE, transferred to a nitrocellulose membrane (Amersham Biosciences) and blotted with primary antibodies overnight at 4°C: anti-pSMAD1/5/8 (1∶1000, Cell signaling, USA), anti-SMAD4 (1∶2000, Cell signaling, USA), anti-CDX2 (1∶1000, Santa Cruz Biotechnology, USA) in 5% non-fat dry milk powder in Tris-buffered saline 0.01%Tween20. Peroxidase-conjugated corresponding secondary antibodies were used and developed with the enhanced chemiluminescent reagent kit (No. RPN2135- Amersham, ECL advance, Western blotting Detection Kit-UK) as per manufacturer's protocol. Quantification of the Western blots was performed using ImageJ software.

### RNA extraction and Reverse transcriptase PCR

RNA was extracted using TRIZOL reagent (Qiagen) according to the manufacturer's protocol. The RNA concentration was quantified by Biophotometer (Eppendorf, Germany). cDNAs were synthesized from RNA in the presence of M-MuLV reverse transcriptase (No. 610600900021730 Merck–Mumbai). Specific primer sequence for MUC5AC was used to amplify gene transcripts. The reaction was run in a thermal cycler (Eppendorf, Germany). The PCR conditions were 5 min of initial denaturation at 95°C and 36 cycles consisting of 45 s of denaturation at 95°C followed by 1 min at 59°C annealing step and 1 min at 72°C elongation steps. The final 10 min incubation at 72°C assured a complete extension of the PCR products. The presence of amplified products were electrophoresed on 1% agarose gel with ethidium bromide and visualized in UV light (Vilber Lourmat, France). The primers (Eurofins scientific, Bangalore, India) used were listed in Table S1 and S2.

### RNA extraction and real-time PCR

Total RNA was extracted using TRIZOL reagent (Qiagen). Reverse transcription was performed with 3 µg of total RNA. BMP2, CDX2, MUC2 and MUC5AC were amplified with SYBR Green (BioRad). GAPDH was used as the endogenous control to normalize the amount of cDNA added to each reaction (ΔCT), and the mean ΔCT of control samples was used as the calibrator to calculate the ΔΔCT. Quantitation of each transcript was by the comparative CT method. In this method, the relative quantity of target mRNA, normalized to the endogenous control and relative to the calibrator, is equal to 2−ΔΔCT. Each experiment was carried out in triplicate at least twice; the results are expressed as means ± SD of representative triplicates. The primers (Eurofins scientific, Bangalore, India) used were listed in Table S3 and S4.

### Alcian blue and PAS staining

Paraffin embedded sections were stained with Alcian blue and PAS staining as mentioned in Linden et al [17]. The slides were then visualized under Axioskope 2 d microscope, Carl Zeiss, Germany.

### Immunohistochemistry and Immunofluorescence

Paraffin embedded samples were serially sectioned at 4 µm, mounted on slides, deparaffinized and dehydrated through a graded series of alcohol. Antigen retrieval was performed by boiling slides for 20 min in 10 mM sodium citrate buffer at pH 6.0 in microwave oven. After cooling the slides were incubated with 3% H_2_O_2_ for 10 min at room temperature. Nonspecific binding was blocked with 3% BSA for 1 hr at room temperature. The slides were incubated with following primary antibodies for overnight at 4°C: anti-pSMAD1/5/8 (1∶200), anti-SMAD4 (1∶200), anti-CDX2 (1∶200) and anti-MUC2 (a kind gift from Dr. Celso A Reis lab, Portugal) (1∶200). The slides were then incubated with corresponding HRP conjugated secondary antibody for 1 hr at room temperature. Slides were then developed with diaminobenzidine and counterstained with Mayer's haematoxylin, rehydrated and mounted. For immunofluorescence, the slides were incubated with primary antibody anti-MUC5AC (a kind gift from Dr. Celso A Reis lab, Portugal) (1∶200) and with secondary antibody conjugated FITC (490/525 nm) (1∶400) and visualized under Axioskope 2 d microscope, Carl Zeiss, Germany.

### Statistical analysis

Data are shown as the mean ± SD. Statistical evaluation was done by unpaired Student's *t* test, and *p*<0.05 was taken as a significant difference.

## Results

### BMP pathway regulates CDX2 expression in *S. dysenteriae* infected rat ileal loop model

**Figure 1:**
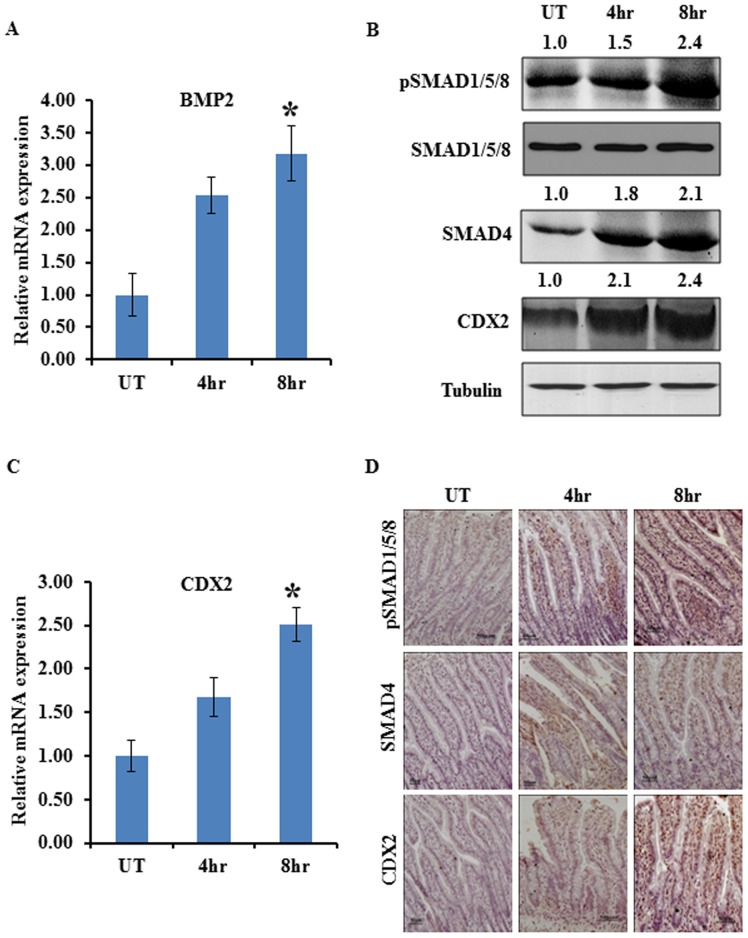
*S. dysenteriae* infected rat ileal loop model stimulates BMP/SMAD pathway. A) Expression of BMP2 transcript level in *S. dysenteriae* infected rat ileal loop tissue at 4 hr and 8 hr and untreated (UT). BMP2 mRNA level was normalized with GAPDH. The values obtained with untreated group are referred to as 1. Data are the mean ± SD. (n = 3). *, p<0.05 B) *S. dysenteriae* infected rat ileal loop lysates were probed for indicated proteins. Tubulin was used as an internal control. C) Expression of CDX2 transcript level in *S. dysenteriae* infected rat ileal loop tissue at 4 hr and 8 hr. CDX2 mRNA level was normalized with GAPDH. The values obtained with untreated (UT) group are referred to as 1. Data are the mean ± SD. (n = 3). *, p<0.05 D) Nuclear pSMAD1/5/8 and cytoplasmic SMAD4 expression were increasing at 4 hr and 8 hr than in untreated (UT) in the villus region of the *S. dysenteriae* infected tissue sections. Nuclear expression of CDX2 was increased in the same conditions as described above. Scale bar represents 50 µM.

To investigate whether *S. dysenteriae* infection activates BMP pathway, we assessed the expression pattern of key elements of this pathway. To analyse this, a total of six male Wistar rats were infected with *S*. *dysenteriae* at ileal section, three rats in each group infected for 4 hr and 8 hr. Three non-infected controls were analyzed for each time points. Hereafter non-infected control is indicated as untreated. The expression of BMP2 was studied by real-time PCR and the expression of pSMAD1/5/8 and SMAD4 which are known as downstream targets of BMP pathway were studied by Western blot and immunohistochemistry in rats infected with *S. dysenteriae* for 4 hr, 8 hr and untreated. A significantly increased expression of BMP2 transcript level was observed at 8 hr than in 4 hr and untreated (Figure 1A). We also found that the expression of phosphorylation of SMAD1/5/8 and SMAD4 were increased by 2.4 and 2.1 fold respectively at 8 hr of infection than at 4 hr and untreated which is generally accepted as readout of an active BMP pathway by Western blot (Figure 1B). In previous studies it has been shown that activation of BMPs regulates CDX2 expression through their downstream targets SMAD4 and pSMAD1/5/8 in AGS cells [18]. Therefore, we next examined CDX2 expression in the *S. dysenteriae* infected rat intestinal tissues at different time intervals. Western blot analysis of CDX2 showed 2.4 fold increase at 8 hr and 2.1 fold increase at 4 hr of infection when compared to untreated (Figure 1B). CDX2 gene expression was measured by RT-PCR (Figure 1C). The CDX2 mRNA level at 8 hr is significantly increased by 1.5 fold when compared to untreated and 4 hr (Figure 1C). Immunohistochemical analysis of pSMAD1/5/8 showed increased nuclear expression at 8 hr than at 4 hr and untreated in villus region and SMAD4 was localized to cytoplasm and nucleus and expression was found increased at 8 hr than at 4 hr and untreated (Figure 1D). This exemplify that BMP pathway becomes more active upon *S. dysenteriae* infection. Immunohistochemical analysis of CDX2 showed increased nuclear expression at 8 hr than at 4 hr and untreated (Figure 1D). These results indicate that BMP/SMAD pathway might regulate CDX2 expression during *S. dysenteriae* infection.

### Induction of MUC2 and differential expression of MUC5AC in *S. dysenteriae* infected rat ileal loop model

**Figure 2:**
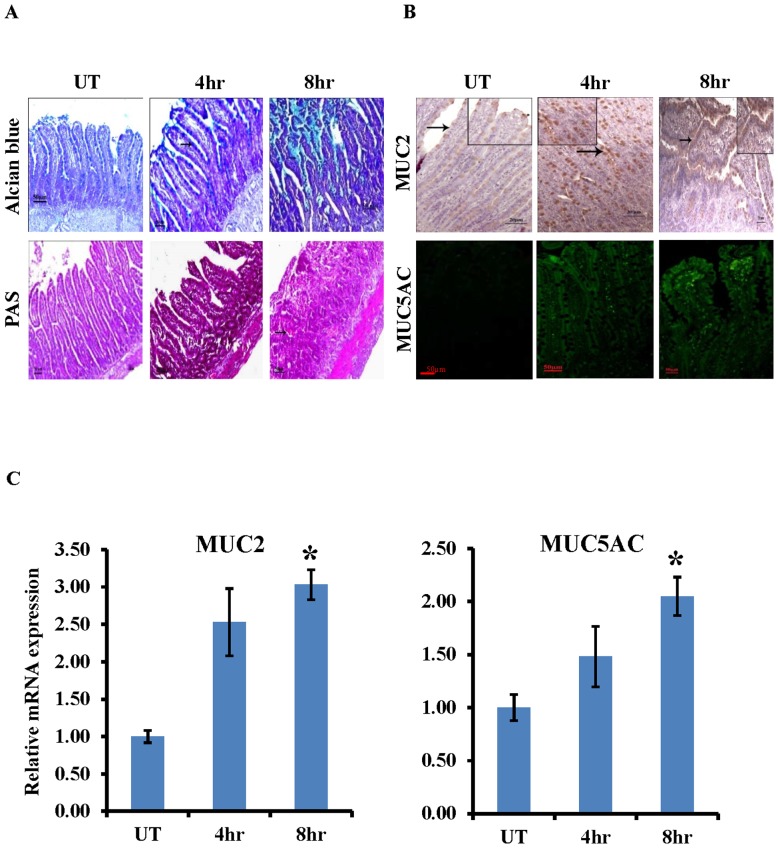
MUC2 and differential expression of MUC5AC in *S. dysenteriae* infected rat model. A) Goblet cells were stained with Alcian blue and PAS staining. Scale bar represents 50 µM. B) Nuclear staining of MUC2 increased at 4 hr and 8 hr of *S. dysenteriae* infected rat ileal tissue sections than untreated (UT) (upper panel). Cytoplasmic MUC5AC expression was induced at 4 hr and 8 hr *S. dysenteriae* infected rat ileal tissue sections. MUC5AC expression was not detected in the untreated group (UT). Scale bar represents 50 µM. C) MUC2 and MUC5AC transcript levels were detected by RT-PCR and were normalized using GAPDH. Data are the mean ± SD. (n = 3). *, p<0.05.

Next we investigated whether activation of BMP-SMAD4 pathway during *S. dysenteriae* infection induces MUC2 and MUC5AC expression. To test our hypothesis we first examined the Alcian Blue/PAS staining in *S. dysenteriae* infected rat ileal section. As shown in Figure 2A Alcian Blue/PAS staining of positive goblet cells was significantly higher at 8 hr and 4 hr of *S. dysenteriae* infection when compared to untreated section. During infection an increase in goblet cell numbers indicates the induction of mucins. To address this we examined the expression of MUC2 in untreated, 4 hr and 8 hr of *S. dysenteriae* infected rat ileal section by immunohistochemistry (Figure 2B upper panel). MUC2 immunoreactivity was present in the nuclei of mucous cells. A significantly higher frequency of MUC2 immunostaining was observed at 8 hr and 4 hr of *S. dysenteriae* infection when compared to untreated section. Next we assessed the expression of MUC5AC by immunofluorescence (Figure 2B lower panel). MUC5AC expression was observed in the cytoplasm of mucous cells at 8 hr and 4 hr of *S. dysenteriae* infection. However, MUC5AC was undetectable in untreated section which indicates that MUC5AC expression in ileum was regulated by *S. dysenteriae.* Furthermore it is important to note that MUC2 and MUC5AC could be produced by the same goblet cells. Interestingly, MUC5AC messenger RNA has been shown to be up-regulated in the intestine during *T. suis* infection in pigs [19]. Therefore, we are curious to know the transcript level of MUC2 and MUC5AC in *S. dysenteriae* infected rat ileal loop model. Now we present data that shows upregulation of MUC2 transcript level during *S. dysenteriae* infection (Figure 2C left panel). Moreover, *S. dysenteriae* infection induces MUC5AC transcript level at 4 hr and 8 hr (Figure 2C right panel). We also confirmed MUC5AC mRNA expression by semi-quantitative assessment and gel image was shown in Figure S1A. Therefore, these data suggest that *S. dysenteriae* upregulates MUC2 expression and also induces differential expression of MUC5AC to increase the porosity of the mucus network.

### BMP-SMAD4-CDX2 signaling is required for mucin induction by *S. dysenteriae*

**Figure 3:**
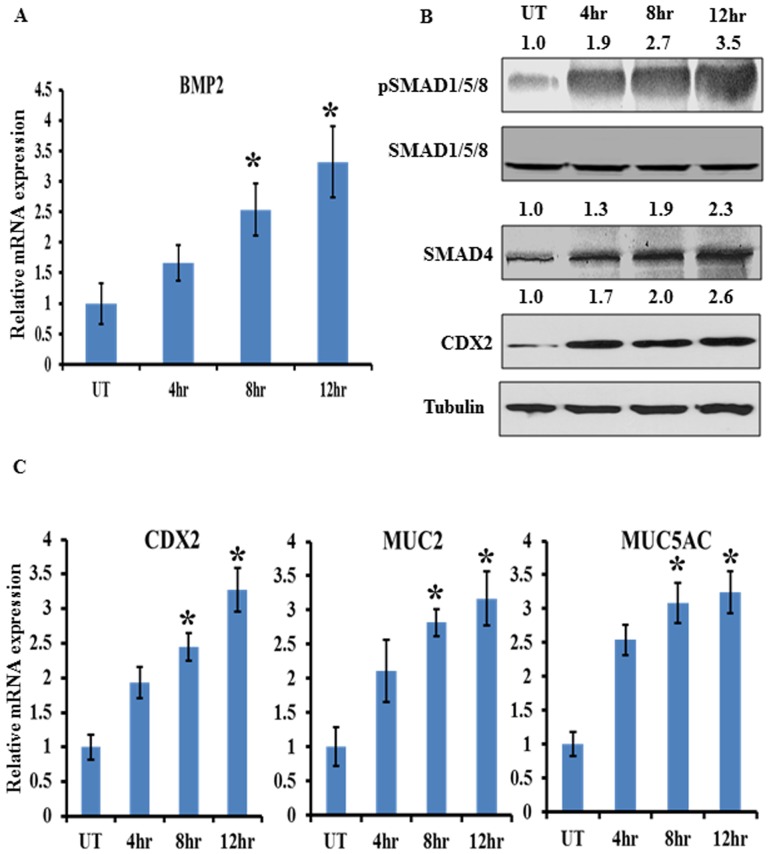
Role of BMP pathway in regulating CDX2 and mucins during *S. dysenteriae* infection in HT29 cell line. A) HT29 cells infected with *S. dysenteriae* at different time intervals were analysed for BMP2 transcript level. The values obtained with untreated cells are referred to as 1. BMP2 mRNA level was normalized with GAPDH. Data are the mean ± SD. (n = 3). *, p<0.05 B) *S. dysenteriae* infected HT29 cells were probed for indicated proteins. Untreated cells were used as control and Tubulin was used as a loading control. C) Expression of CDX2, MUC2 and MUC5AC transcript levels were determined in *S. dysenteriae* infected HT29 cells. The mRNA levels were normalized using GAPDH. Data are the mean ± SD. (n = 3). *, p<0.05.

Our *S. dysenteriae* infected rat ileal loop model suggests that BMP pathway might induce the MUC2 and MUC5AC transcription. We next elucidated the role of BMP pathway in *S. dysenteriae* infected *in vitro* model. To test that we co-cultured HT29 cells with *S. dysenteriae* strain at different time intervals. We studied the expression of BMP2 by real time PCR and pSMAD1/5/8, SMAD4 expression by Western blot analysis which is generally accepted as readout of an active BMP pathway. We observed that BMP2, pSMAD1/5/8, and SMAD4 and CDX2 were significantly increased in a time dependent manner (Figure 3A–B). These data suggest that the pathway becomes more active upon *S. dysenteriae* infection and replicates our *in vivo* data. CDX2, MUC2 and MUC5AC transcriptional level were assessed by real time PCR in the same cells and found that CDX2 and MUC2 were significantly upregulated and MUC5AC was differentially expressed in a time dependent manner (Figure 3C). We also confirmed MUC5AC mRNA expression by semi-quantitative assessment and gel image was shown in Figure S1B. Collectively, we conclude from these data that *S. dysenteriae* regulates BMP pathway mediated mucin expression *in vivo* and *in vitro.*

### SMAD4 acts upstream of CDX2 in regulation of mucin induction by *S. dysenteriae*

**Figure 4:**
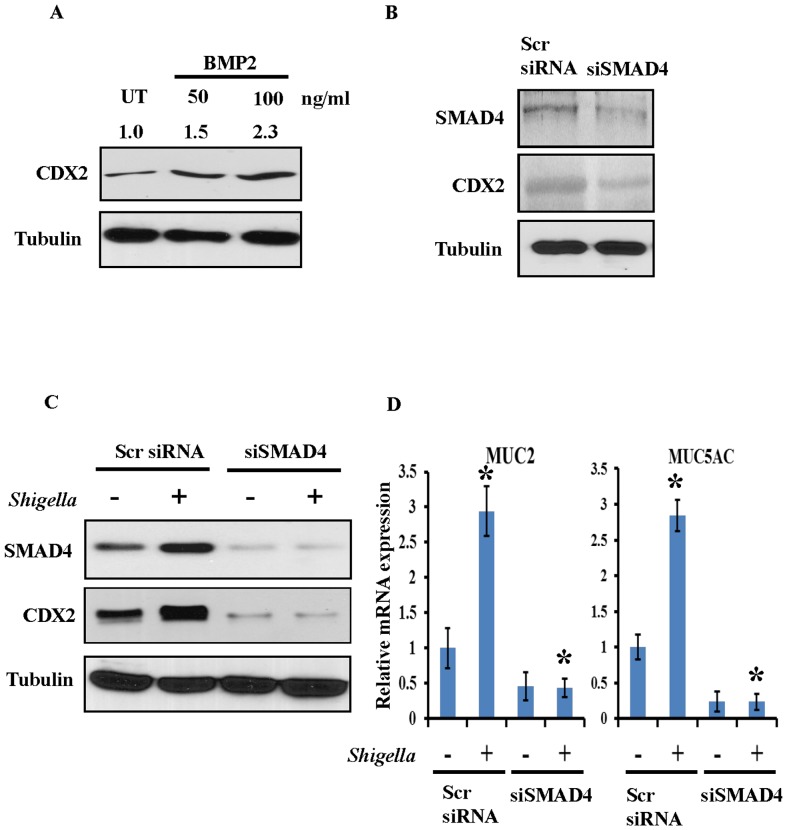
SMAD4 regulates CDX2 and mucin. A) Different concentration of BMP2 ligand were stimulated in HT29 cells and probed for CDX2 expression. Vehicle treated cells were used as control. B) SMAD4 silenced HT29 cells were probed for indicated proteins. Tubulin was used as a loading control. C) Scrambled and SMAD4 silenced cells were infected with *S. dysenteriae* and probed for indicated proteins. Tubulin was used as a loading control. D) MUC2 and MUC5AC transcript levels were detected in *S. dysenteriae* infected scrambled and SMAD4 silenced cells. Data are the mean ± SD. (n = 3). *, p<0.05.

To assess whether BMP pathway regulates CDX2, HT29 cells were treated with BMP2 in the culture medium. It is clear from our result that BMP2 ligand induces CDX2 expression (Figure 4A) in a dose dependent manner. Further to investigate whether BMP pathway upregulates CDX2 expression, SMAD4 an intermediate of BMP pathway was silenced using SMAD4 siRNA and respective scrambled were included. The knockdown efficiency of SMAD4 siRNA was shown in Figure 4B upper panel. Our result revealed that CDX2 was down regulated in SMAD4 silenced cells when compared with scrambled (Figure 4B second panel). Then, *S. dysenteriae* was infected in scrambled and SMAD4 silenced HT29 cells. Our result showed impairment of CDX2 upregulation by *S. dysenteriae* when compared with scrambled infected cells (Figure 4C). Thus our result indicates that CDX2 was upregulated through BMP pathway during *S. dysenteriae* infection. We next investigated whether SMAD4 regulates MUC2 and MUC5AC induction during *S. dysenteriae* infection. As shown in Figure 4D MUC2 and MUC5AC transcriptional levels were reduced in SMAD4 silenced cells whereas *S. dysenteriae* infection did not have any further effect. We also confirmed by semi-quantitative assessment that *S. dysenteriae* infection in SMAD4 silenced cells did not induce MUC5AC mRNA expression and gel image was shown in Figure S1C. These data suggests that BMP pathway is critically involved in regulating MUC2 and MUC5AC induction during *S. dysenteriae* infection.

### SMAD4 directly regulates *S. dysenteriae* induced MUC5AC mucin transcription

**Figure 5:**
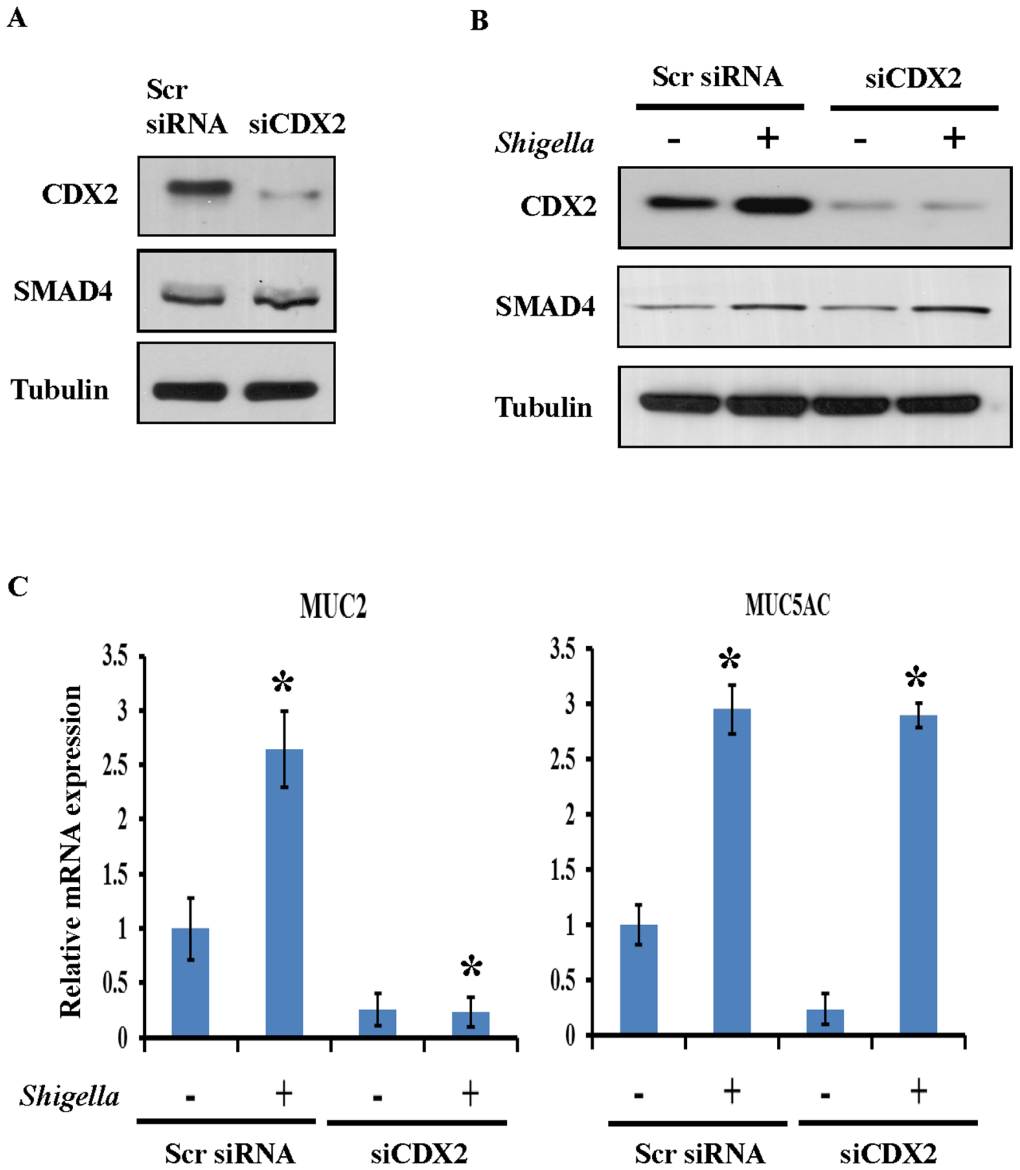
*S. dysenteriae* induced differential expression of MUC5AC directly by SMAD4. A) Scrambled and CDX2 silenced cells were probed for indicated proteins. Tubulin was used as a loading control. B) Scrambled and CDX2 silenced cells were infected with *S. dysenteriae* and probed for indicated proteins. Tubulin was used as a loading control. C) MUC2 and MUC5AC transcript levels were detected in *S. dysenteriae* infected scrambled and CDX2 silenced cells. Data are the mean ± SD. (n = 3). *, p<0.05.

Because of the important role of CDX2 in mediating bacteria induced activation of mucins, we next evaluated the role of CDX2 in MUC2 and MUC5AC induction by *S. dysenteriae*. To further confirm the role of CDX2 in mucin induction, knockdown experiments using CDX2 siRNA was performed in HT29 cells. The knockdown efficiency of siCDX2 was shown in Figure 5A (upper panel). SMAD4 protein level was not changed in CDX2 silenced cells indicates that SMAD4 is upstream of CDX2. To further determine whether SMAD4 acts upstream of CDX2 in regulation of mucin induction scrambled and CDX2 silenced cells were infected with *S. dysenteriae*. As we expected *S. dysenteriae* increased CDX2 expression in scrambled cells and did not have any further effect on the CDX2 silenced cells (Figure 5B). Interestingly, *S. dysenteriae* infection increased the SMAD4 expression in scrambled as well as in siCDX2 cells which indicates that CDX2 function downstream of SMAD4. In the same experiment we evaluated the MUC2 and MUC5AC transcript level by real time PCR analysis. As shown in Figure 5C (left panel) we observed that inhibition of CDX2 by siRNA significantly suppressed MUC2 transcript level where as *S. dysenteriae* infection did not have any further effect. In contrast MUC5AC transcript level was induced by *S. dysenteriae* infection (Figure 5C right panel) and we also confirmed by semi-quantitative assessment and gel image was shown in Figure S1D. These data indicates that SMAD4 directly regulates MUC5AC induction whereas MUC2 expression is regulated through CDX2.

## Discussion

**Figure 6:**
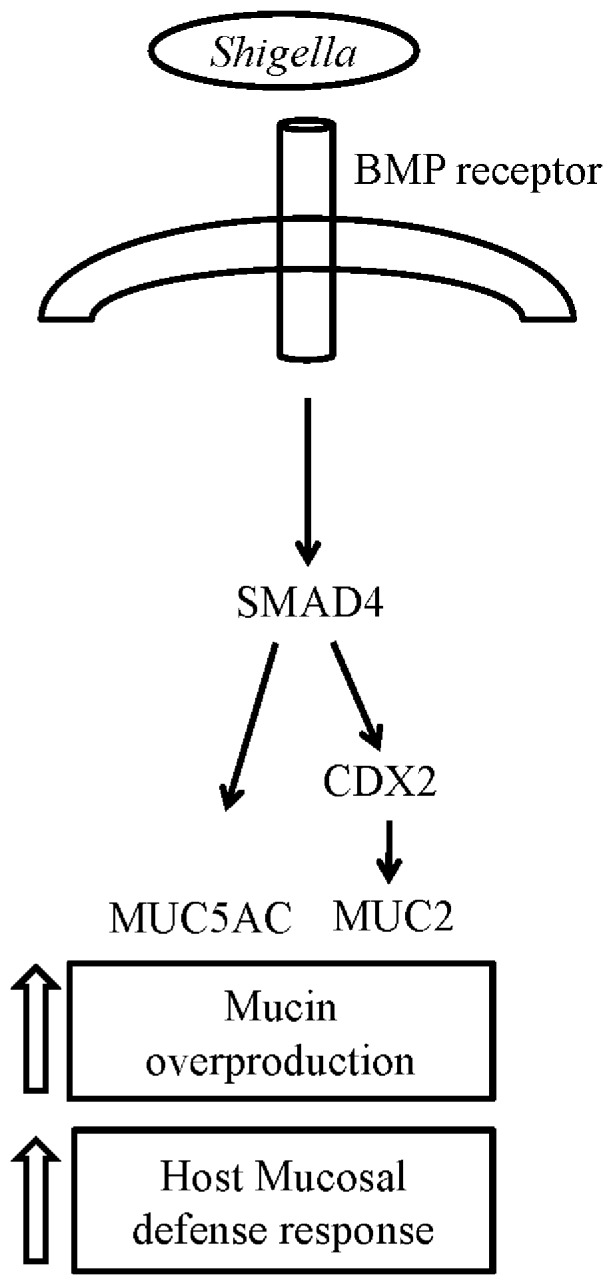
Schematic representation of mucin gene induction by *Shigella*. As indicated, BMP-SMAD pathway induces MUC2 gene expression through the CDX2 transcription factor whereas SMAD4 directly influences MUC5AC gene. Our data thereby unveil a complex signaling mechanism underlying mucin gene induction by *Shigella*.

The mucus layer is an important component of host defense against microbial infection of the intestinal epithelium. The goal of the current study provides insight into mechanism of mucin gene expression driven by inflammation. Here we showed *in vivo* and *in vitro* model that the *S. dysenteriae* stimulates BMP pathway to upregulate MUC2 and MUC5AC mucin gene expression. *S. dysenteriae* activated BMP pathway modulates expression of CDX2 transcription factor which is required for the induction of MUC2 gene. Interestingly we also demonstrated that direct involvement of the SMAD4 in the transcriptional regulation of the MUC5AC gene during *S. dysenteriae* infection. Thus our studies provide novel insights into the molecular mechanisms underlying the tight regulation of mucin overproduction in the pathogenesis of *S. dysenteriae* infectious diseases that may lead to development of new therapeutic strategies (Figure 6).

The BMP pathway have been well studied and are known to be important signaling pathway diversely involved in embryonic development, angiogenesis and inflammatory stimuli [20] but little is known about their role in regulating mucin gene expression. In the present study, we identified a critical role of BMP pathway in mucin gene induction by *S. dysenteriae*. We also found that transcription factor CDX2 was regulated by BMP pathway during *S. dysenteriae* infection. Our results are also in accordance with a previous observation that activation of BMP pathway is associated with CDX2 expression during *Helicobacter pylori* infection in both *in vivo* and *in vitro* model [12]. On the other hand there is also evidence showing that activated BMP pathway associated with CDX2 expression in different models of esophagitis and Barrett's esophagus [21], [22]. Here, we reinforce the previously identified BMP-CDX2 interaction, which we illustrate for the first time in *S. dysenteriae* infected rat ileal loop and *in vitro* model.

Altered expression of CDX2 can disrupt the mucosa in protecting the host against its luminal components in the intestine, resulting in an increased expression of inflammatory mediators leading to colonic inflammation as observed in inflammatory bowel disease (IBD) [23]. Recent studies showed that CDX2 regulates MUC2 expression [24]–[26]. Ikeda *et al* found that bacterial components induce CDX2 expression followed by MUC2 expression, both *in vivo* and *in vitro* [27]. We also showed in our *in vivo* and *in vitro* model that CDX2 acts as a major transcriptional regulator of MUC2 gene. These suggest that in *S. dysenteriae* infection BMP-SMAD4 pathway promotes CDX2 expression which is capable of inducing MUC2. Growth factors mediated activation of SMAD transcription factors [28] form either SMAD2-SMAD4 or SMAD3-SMAD4 complexes to translocate into the nucleus where they bind to the promoter of the target gene to activate transcription. However, once bound to the promoter, SMAD4 may interact with other factors like CDX2 and Sp1 to activate transcription [29]. Moreover, another study suggests that CDX2 also binds SMAD3 to regulate its activity [30]. Based on these previous studies [12], [18], [29], [30] and also from our SMAD4 silencing data it clearly suggests that CDX2 is regulated by BMP pathway. Therefore the data presented in this article indicates that SMAD4 might interact with CDX2 to induce MUC2 transcription. To the best of our knowledge, this is the first description of CDX2 induced MUC2 gene expression upon infection with *S. dysenteriae*.

The MUC2 rich mucus layer is the first host-defense barrier that functions as the main interface between the host and its luminal microbiota [31]. MUC2 plays a significant but partial role in protection against *T.muris* infection whereas MUC5AC expression correlates with worm expulsion in resistant mice even in the absence of the predominant baseline intestinal secreted mucin MUC2 [5]. MUC5AC, which is a product of normal gastric mucosa is absent in normal colon but induced in intestine during *T.suis, Nippostringylus brasiliensis* and *Trichinella spiralis* infectious models [19], [32], [33]. In consistent with these data *S. dysenteriae* also induces MUC5AC expression by direct influence of BMP pathway in rat ileal loop model. The MUC5AC promoter contains binding sites for the SMAD4 transcription factor throughout its sequence [34]. In the present study we found that *S. dysenteriae* infection in SMAD4 silenced cells downregulates the MUC2 and MUC5AC gene whereas CDX2 silenced cells induces MUC5AC expression and inhibits MUC2 gene. Together, these studies suggest that SMAD4-CDX2 induces MUC2 expression and SMAD4 directly influences MUC5AC expression. Our findings elucidate that inflammatory transcription factors such as SMAD4 and CDX2 are required for achieving mucin overproduction during *S. dysenteriae* infection. Thus for the first time we identified MUC2 and MUC5AC gene expression as a direct and critical mediator of BMP pathway during *S. dysenteriae* infection which might promote bacterial expulsion.

MUC5AC within the mucus layer may influence the biochemical properties of the mucus gel and thus it might facilitate *S. dysenteriae* expulsion. MUC5AC increases the viability of the bacteria by increasing the porosity of the mucus network. Furthermore MUC5AC not only play an important and indispensable role in worm expulsion it also functions as the key immune effector molecule [33]. Moreover, MUC2 and MUC5AC not just as a structural component of the mucus barrier but also act as a crucial effector molecule during infections of the intestine. Although, we and others showed the relationship between bacterial infection and overexpression of MUC2 and MUC5AC in the intestine, respiratory tract and middle ear infection [8], [35], [36] but the transcription factor involved in the mucin gene induction was not determined during *S. dysenteriae* infection. In our previous studies we reported that *S. dysenteriae* infection stimulates the crosstalk between IL-1β and AKT signaling which promotes TFF3 expression to induce MUC5AC expression [9]. Additionally, we now show that *S. dysenteriae* infected rat ileal loop and *in vitro* model activates BMP pathway to promote CDX2 expression which induce MUC2 gene whereas SMAD4 directly stimulates MUC5AC gene transcription. The transcription factor CDX2 controls the expression of a number of downstream genes, some of which being thought to play a key role in inflammation. For instance CDX2 is a positive regulator of the TFF3 gene [37]. Therefore, it's likely that BMP pathway might cross talk with the other signaling pathways to induce mucin gene expression.

In the present study, we show that MUC2 and MUC5AC mucin expression is tightly regulated in response to *S. dysenteriae* via BMP signaling pathway. Furthermore, we found that *S. dysenteriae* activates BMP-SMAD4 pathway which upregulates MUC2 gene transcription by inducing CDX2 expression whereas SMAD4 directly influences MUC5AC gene expression. It suggests that BMP pathway may play more important role in mucin activation in response to *S. dysenteriae* infection. Future studies will help to unravel the associated mechanisms of interaction between BMP receptor and *S. dysenteriae.* Nonetheless, our studies unveil a novel complex signaling mechanism involved in regulation of host mucosal defense in response to *S. dysenteriae* infection and it may help to develop a new therapeutic strategy.

## Supporting Information

Figure S1 ***S. dysenteriae*** **infection induces MUC5AC expression** ***in vivo*** **and** ***in vitro*** **.** A) Gel image and semi-quantitative assessment of MUC5AC mRNA expression in *S. dysenteriae* infected rat ileal loop sections detected by RT-PCR. GAPDH expression was used as an internal control. B) Gel image and semi-quantitative assessment of MUC5AC mRNA expression in *S. dysenteriae* infected HT29 cells detected by RT-PCR. GAPDH expression was used as an internal control. C) RT-PCR analysis showing that *S. dysenteriae* infection induces MUC5AC expression in scrambled siRNA cells whereas it did not have any further effect in SMAD4 silenced cells. GAPDH expression was used as an internal control. D) RT-PCR analysis showing that *S. dysenteriae* infection induces MUC5AC expression in scrambled siRNA cells as well as in CDX2 silenced cells. GAPDH expression was used as an internal control.
(TIFF)

Table S1 **Semi-quantitative PCR primers for Rat intestinal tissue.**
(DOC)

Table S2 **Semi-quantitative PCR primers for Cell line.**
(DOC)

Table S3 **List of Real-time Primers for Rat Intestinal tissue.**
(DOC)

Table S4 **List of Real-time Primers for Cell line.**
(DOC)
