# Supplementary figures and images for: Shigella dysenteriae Modulates BMP Pathway to Induce Mucin Gene Expression In Vivo and In Vitro
Source: PLoS One. 2014 Nov 3;9(11):e111408. doi: 10.1371/journal.pone.0111408 (PMC4218725; doi:10.1371/journal.pone.0111408)

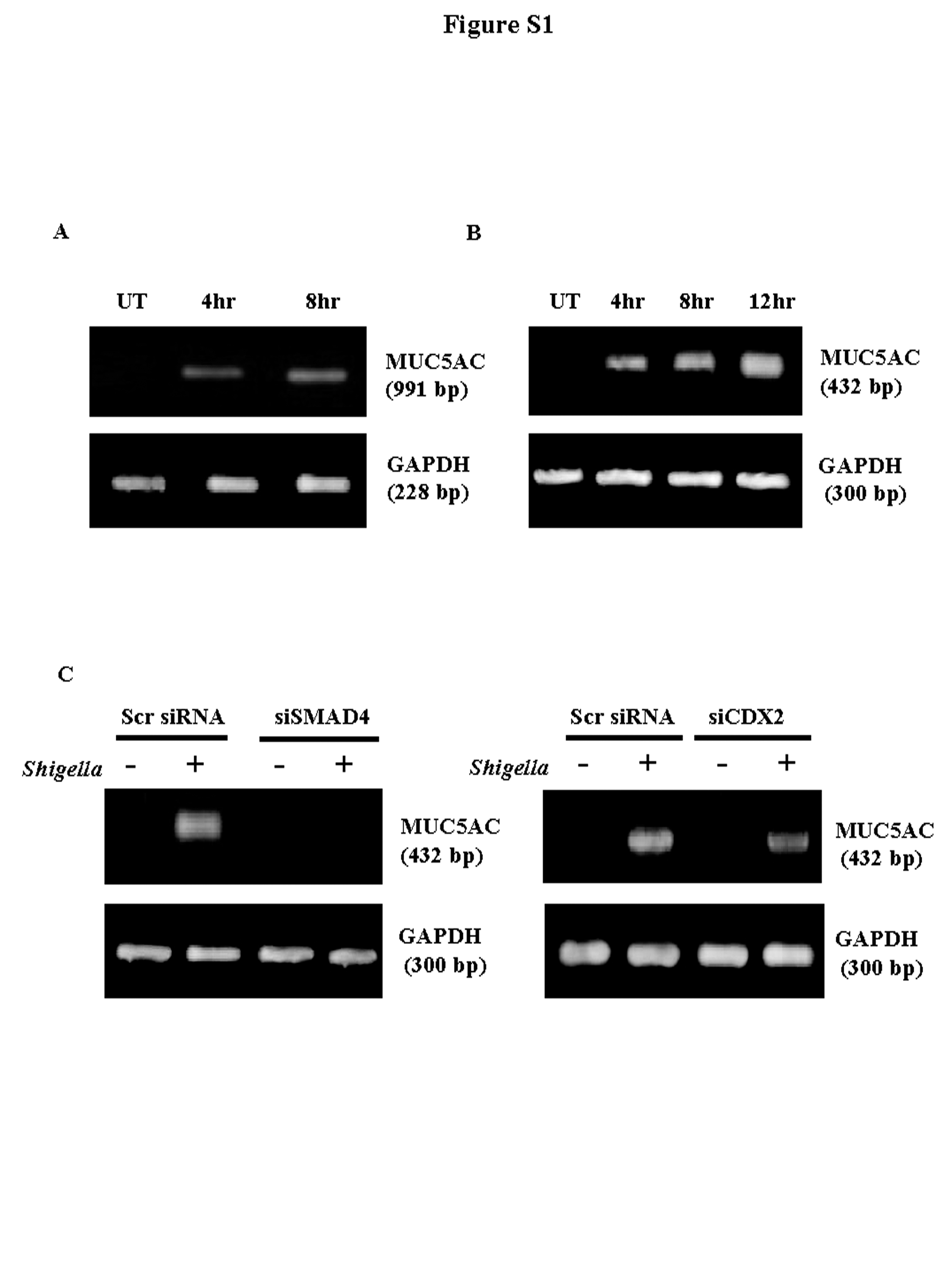

Supplement: Figure S1 — S. dysenteriae infection induces MUC5AC expression in vivo and in vitro . A) Gel image and semi-quantitative assessment of MUC5AC mRNA expression in S. dysenteriae infected rat ileal loop sections detected by RT-PCR. GAPDH expression was used as an internal control. B) Gel image and semi-quantitative assessment of MUC5AC mRNA expression in S. dysenteriae infected HT29 cells detected by RT-PCR. GAPDH expression was used as an internal control. C) RT-PCR analysis showing that S. dysenteriae infection induces MUC5AC expression in scrambled siRNA cells whereas it did not have any further effect in SMAD4 silenced cells. GAPDH expression was used as an internal control. D) RT-PCR analysis showing that S. dysenteriae infection induces MUC5AC expression in scrambled siRNA cells as well as in CDX2 silenced cells. GAPDH expression was used as an internal control. (TIFF) [file pone.0111408.s001.tiff]
