# Supplementary material for: Shigella dysenteriae Modulates BMP Pathway to Induce Mucin Gene Expression In Vivo and In Vitro
Source: PLoS One. 2014 Nov 3;9(11):e111408. doi: 10.1371/journal.pone.0111408 (PMC4218725; doi:10.1371/journal.pone.0111408)
Supplement: Table S1 — Semi-quantitative PCR primers for Rat intestinal tissue. (DOC) [file pone.0111408.s002.doc]

Table S1: Semi-quantitative PCR primers for Rat intestinal tissue

| **S.NO** | **Primer Name** | **Forward (5’ to 3’)** | **Reverse (5’ to 3’)** |
| --- | --- | --- | --- |
| 1. | MUC5AC | 5’- CCCTTGAGGACACTCGGAAA-3’ | 5’- GGGTTGGAGCAGGTGTCTAC-3’ |
| 2. | GAPDH | 5’ – AGCCATGTACGTAGCCATCC-3’ | 5’ –CTCTCAGCTGTGGTGGTGAA-3’ |
