# Supplementary material for: Shigella dysenteriae Modulates BMP Pathway to Induce Mucin Gene Expression In Vivo and In Vitro
Source: PLoS One. 2014 Nov 3;9(11):e111408. doi: 10.1371/journal.pone.0111408 (PMC4218725; doi:10.1371/journal.pone.0111408)
Supplement: Table S3 — List of Real-time Primers for Rat Intestinal tissue. (DOC) [file pone.0111408.s004.doc]

| **S.NO** | **Primer Name** | **Forward (5’to 3’)** | **Reverse (5’to 3’)** |
| --- | --- | --- | --- |
| 1. | BMP2 | 5’-GGAAAACTTCCCGACGCTTCT-3’ | 5’-CCTGCATTTGTTCCCGAAAA-3’ |
| 2. | CDX2 | 5’- ATCACCATCAGGAGGAAAGC-3’ | 5’- TTTTCCTTTCCTTGGCTCTG-3’ |
| 4. | MUC2 | 5’- TCTTCAGCTTCCCCAATCAC-3’ | 5’- GGACCAATTGAAGACCTGGA-3’ |
| 5. | MUC5AC | 5’- GGCTGGTCTTCATGTGGAAT-3’ | 5’- CTCAAGGGGTGTCAGCCTAA-3’ |
| 6. | GAPDH | 5’- ATGGTGAAGGTCGGTGTGA-3’ | 5’- GGTAGAGTCATACTGGAACATGTAGA-3’ |

**Table S3: List of Real-time Primers for Rat Intestinal tissue**
