# Supplementary material for: Shigella dysenteriae Modulates BMP Pathway to Induce Mucin Gene Expression In Vivo and In Vitro
Source: PLoS One. 2014 Nov 3;9(11):e111408. doi: 10.1371/journal.pone.0111408 (PMC4218725; doi:10.1371/journal.pone.0111408)
Supplement: Table S4 — List of Real-time Primers for Cell line. (DOC) [file pone.0111408.s005.doc]

| **S.NO** | **Primer Name** | **Forward (5’to 3’)** | **Reverse (5’to 3’)** |
| --- | --- | --- | --- |
| 1. | BMP2 | 5’- CTCAGGTCAGCCGGGCTCA-3’ | 5’- GTTCTTCCAAAGATTCTTCATGC-3’ |
| 2. | CDX2 | 5’- TTCACTACAGTCGCTACATCACC-3’ | 5’- TTGTTGATTTTCCTCTCCTTTGC-3’ |
| 3. | MUC2 | 5’- GCTGTCCCTTCTACTGGTGT-3’ | 5’- GTTGAGCAGGGTGTTGTTGT-3’ |
| 4. | MUC5AC | 5’- TGTTTCTCCCCATCATTCCAGA-3’ | 5’- ATCTGGATGCCTGAGTTGTCC-3’ |
| 5. | GAPDH | 5’- GAAGGTGAAGGTCGGAGTC-3’ | 5’ – GAAGATGGTGATGGGATTTC-3’ |

**Table S4: List of Real-time Primers for Cell line**
